# MicroRNA-124 Reduces Arsenic-induced Endoplasmic Reticulum Stress and Neurotoxicity and is Linked with Neurodevelopment in Children

**DOI:** 10.1038/s41598-020-62594-8

**Published:** 2020-04-03

**Authors:** Hae-Ryung Park, Ryan Sun, Ronald A. Panganiban, David C. Christiani, Quan Lu

**Affiliations:** 1 Program in Molecular and Integrative Physiological Sciences, Departments of Environmental Health, and Genetics & Complex Diseases, Harvard T.H. Chan School of Public Health, Boston, Massachusetts 02115 USA; 2 Department of Biostatistics, Harvard T.H. Chan School of Public Health, Boston, Massachusetts 02115 USA

**Keywords:** Cell biology, Genotype, Biomarkers

## Abstract

Arsenic (As) exposure adversely affects neurodevelopment in children. Accumulation of misfolded proteins in cells exposed to As leads to endoplasmic reticulum (ER) stress response, which, if not relieved, results in cell death. Despite the potential role of ER stress for As-induced neurotoxicity, the underlying mechanisms remain poorly understood. Here we aimed to investigate the roles of microRNA(miR)-124, a novel ER stress suppressor, in As-induced ER stress response and cytotoxicity in neural cells. We further aimed to link these *in vitro* findings to neurodevelopmental outcomes in children who were exposed to As. Using Quantitative RT-PCR and Cyquant assay, we showed that miR-124 protects against As-induced cytotoxicity in neural cells with concomitant suppression of As-induced ER stress. In addition, As-induced cytotoxicity was exacerbated in miR-124 knockout cells generated by CRISPR-based gene editing compared scramble control. Furthermore, we identified two miR-124 SNPs rs67543816 (p = 0.0003) and rs35418153 (p = 0.0004) that are significantly associated with a mental composite score calculated from the Bayley Scales of Infant Development III in Bangladesh children. Our study reveals As-induced ER stress as a crucial mechanism underlying the toxic effects of As on neural cell function and neurodevelopment and identifies miR-124 as a potential preventative and therapeutic target against detrimental effects of As exposure in children.

## Introduction

Exposure to arsenic (As) has been a serious public health concern because it has been attributed to myriad human diseases. Especially, As exposure during early brain development has persistent effects on neurocognitive function. Multiple epidemiologic studies report that As exposure is associated with neurocognitive deficits in children^1–10^. For example, maternal and child urinary As levels inversely predicted performance IQ and verbal IQ, respectively, among Bangladesh children^2^. Furthermore, elevated drinking water As levels adversely impacted IQ in children^10^. Animal studies also confirmed the adverse impact of As on neurobehavioral and cognitive functions^11–15^. Although epidemiological and animal studies have clearly established the neurotoxicity of As, the molecular mechanisms by which As impairs neuronal functions remain poorly understood.

As can cause protein misfolding and induces stress response in the endoplasmic reticulum (ER), a multi-functional organelle essentiall for synthesis, folding, and processing of proteins^16–18^. ER stress has arisen as a potential mechanism for As-induced neurotoxicity^19–23^. A toxic inorganic species arsenite (As(III)) can react with thiols, leading the inhibition of protein function and misfolding^24,25^. As-induced ER stress then triggers unfolded protein response (UPR) to restore ER homeostasis, mediated by three ER transmembrane receptors, including inositol-requiring protein-1 (IRE1), protein kinase RNA-like ER kinase (PERK), and activating transcription factor 6 (ATF6)^16^. However, excessive ER stress, if not resolved, leads to cell death^26^, which is mainly mediated through the apoptotic transcription factor CHOP (C/EBP homologous protein, DDIT3). As exposure has shown to induce apoptosis with increased expression of ER stress markers in a mouse neuroblastoma cell line and rat brain^19,23^. In addition, As-induced ER stress and apoptosis in the rat hippocampus was associated with learning and memory impairment^27^, suggesting potential roles of As-induced ER stress in the development of adverse neurocognitive outcomes.

**Figure 1:**
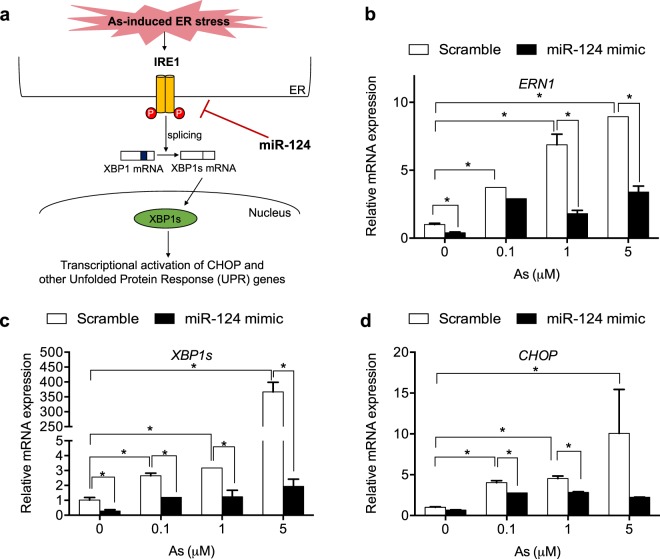
Mir-124 suppresses As-induced IRE1 ER stress/UPR pathway in human neural cells (**a**) Schematic of As-induced activation of the IRE1 ER stress/UPR pathway. Mir-124 specifically down-regulates IRE1 expression to suppress XBP1 splicing and CHOP activation^35^. (**b–d**) Effect of miR-124 on As-induced ER stress response. SH-SY5Y neuroblastoma cells were transfected with scramble or miR-124 mimic (10 pmol/well), then exposed to As for 4 h. Expression of *ERN1* (gene encoding IRE1), *XBP1s* (spliced XBP1), and *CHOP* was measured by qRT-PCR. N = 3 experiments. All error bars represent the standard error of the mean of three biologic replicates. *p < 0.05.

MicroRNAs are small non-coding RNAs (∼21–25 nucleotides in length) that mostly binds to the 3′ untranslated region (UTR) of target genes and suppress their expression by degrading targeting messenger RNAs (mRNAs) or by interfering translation^28,29^. Growing evidence shows that microRNAs are key players in the regulation of ER stress/UPR signaling^30–34^. We previously identified microRNA (miR)-124-3, via a genome-wide CRISR-based screen, as a suppresser of ER stress-induced apoptosis^35^. MiR-124 directly targets the IRE1 pathway among three UPR branches (Fig. 1A). Interestingly, MiR-124 is the most abundant microRNA in the brain and plays important roles in neurogenesis, neuronal differentiation, and proliferation^36–39^. While our findings implicate microRNA-124 in the regulation of ER stress response, it is not known whether such regulation contributes to the role of miR-124 in the CNS and neural cells, and ultimately affect neurodevelopmental outcomes in children. In this study, we investigated the role of microRNA-124 in As-induced ER stress and cytotoxicity in multiple types of neural cells and examined the genetic association of miR-124 with adverse neurodevelopmental outcomes in children exposed to As. We showed that miR-124 protects against As toxicity in human neural cells *in vitro*, with concomitant down-regulation of As-induced ER stress response. Furthermore, we showed that genetic polymorphisms of miR-124-3 were associated with neurocognitive outcomes in children included in an epidemiological cohort.

## Results

### MiR-124 suppresses As-induced ER stress response genes in neural cells

**Figure 2:**
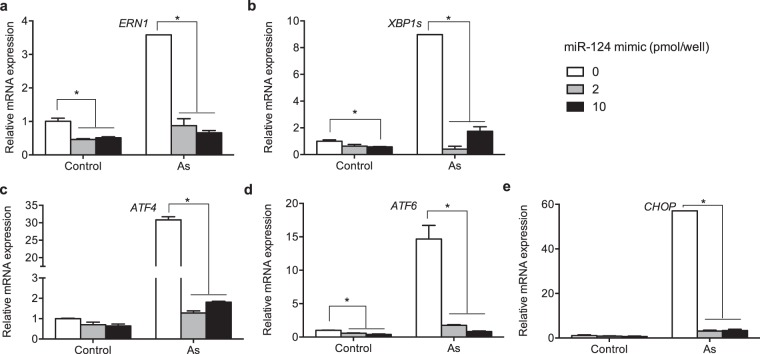
Mir-124 suppresses As-induced activation of three UPR pathways in human neural cells (**a–e**) Effect of miR-124 on As-induced ER stress response. ReNcell Cx neuroprogenitor cells were transfected with scramble or miR-124 mimic (5 pmol or 10 pmol/well), then exposed to 10 μM As for 4 h. Expression of *ERN1* (gene encoding IRE1), *XBP1s* (spliced XBP1), *ATF4, ATF6* and *CHOP* was measured by qRT-PCR. N = 3 experiments. All error bars represent the standard error of the mean of two biologic replicates. *p < 0.05.

We previously reported that miR-124 suppresses As-induced ER stress response and cell death in embryonic kidney HEK293T cells, by direct binding to the 3′UTR of IRE1 gene (*ERN1*) and subsequent down-regulation of the IRE1 pathway (Fig. 1A)^35^. Because miR-124 is the most abundant microRNA in the brain^40,41^ and plays important roles in neural differentiation and proliferation^36–39^, we investigated the role of miR-124 in neural cells in response to As exposure. As shown in Fig. 1B, As treatment upregulated expression of *ERN1* in a concentration dependent manner in neuroblastoma SH-SY5Y cells, indicating that As-induced ER stress activates the IRE1 UPR pathway. Because activation of IRE1 leads to endonucleolytic cleavage of X-box binding protein-1 (XBP-1) mRNA, generating *XBP1s* that encodes a potent transcriptional activator XBP1s^16^, we next measured expression of *XBP1s* in As-treated SH-SY5Y cells. As shown in Fig. 1C, As treatment resulted increased expression of *XBP1s* (Fig. 1C), indicating the increased XBP1 slicing by IRE1 activation. Because binding of XBP1s to the promoter region of *CHOP* gene leads to upregulation of pro-apoptotic transcription factor CHOP, we also investigated the effect of As treatment on *CHOP* expression in the cells. Figure 1D shows that As increased expression of *CHOP* in a concentration-dependent manner in SH-SY5Y cells. These data suggest that As-induced ER stress activates the IRE1 pathway and pro-apoptotic CHOP in neural cells. To test the role of miR-124 in the regulation of As-induced ER stress in neural cells, SH-SY5Y cells were transfected with miR-124 synthetic mimic or scramble control, then exposed to As. Overexpression of miR-124 by mimic transfection significantly down-regulated expression of *ERN1* in the absence or presence of As (Fig. 1B), indicating that miR-124 targets IRE1 to suppress As-induced ER stress response. Similarly, miR-124 mimic transfection also leads to suppression of *XBP1s* and *CHOP* (Fig. 1C,D). These results indicate that miR-124 suppresses As-induced ER stress response in the SH-SY5Y neural cells. To test whether miR-124 suppresses PERK or ATF6 pathways, we examined the effect of miR-124 mimic on the expression of *ATF4* and *ATF6* as well as *ERN1, XBP1s*, and *CHOP* in ReNcell Cx neuroprogenitor cells. As shown in Fig. 2, miR-124 mimic transfection significantly suppressed mRNA expression of *ERN1, XBP1, ATF4, ATF6*, and *CHOP* implicating that miR-124 suppresses PERK and ATF6 UPR pathways in addition to IRE1 pathway. Furthermore, miR-124 also suppressed thapsigargin or tunicamycin-induced ER stress in the cells, indicating that miR-124 is a suppressor of ER stress induced by multiple inducers (Supplementary Fig. 1). Because PERK is the major regulator of CHOP, we tested how pretreatment with ISRIB, a potent PERK inhibitor modulates the effect of miR-124. As shown in Supplementary Fig. 2A, ISRIB downregulated As-induced *ATF4* expression in the presence or absence of miR-124 mimic. ISRIB treatment alone significantly downregulated As-Induced CHOP expression and its effect was comparable to the effect of miR-124 mimic (Supplementary Fig. 2B), indicating that miR-124-mediated CHOP suppression is mainly by suppressing PERK pathway. When cells were transfected with miR-124 following ISRIB pretreatment, CHOP expression was even more suppressed compared with mimic alone (Supplementary Fig. 2B), suggesting that miR-124 suppresses CHOP through multiple mechanisms.

### MiR-124 is protective against As toxicity in neural cells

**Figure 3:**
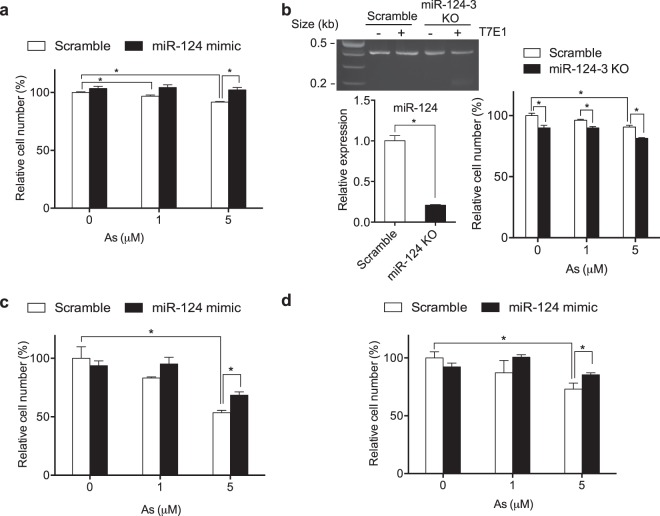
MiR-124 protects against As-induced cytotoxicity in human neural cells. (**a**) Effect of miR-124 overexpression on cell viability in SH-SY5Y cells. (**b**) Effect of miR-124 knockout on cell viability in SH-SY5Y cells. *Left top*. Validation of miR-124 knockout by T7E1 assay. *Left bottom*. Validation of miR-124 knockout by qRT-PCR. *Right*. Scrambled or miR-124 knockout SH-SY5Y cells were treated with As for 48 h. (**c**) Effect of miR-124 overexpression on cell viability in human neural stem cells (hNSCs) (**d**) Effect of miR-124 overexpression on cell viability in ReNcell Cx neuro-progenitor cells. For mimic experiments, cells were transfected with scramble or miR-124 mimic (10 pmol/well) for 24 h, then exposed to As for 48 h. Cell viability was measured by CyQuant assay. N = 3 experiments. All error bars represent the standard error of the mean of three biologic replicates. *p < 0.05.

Based on our data showing the protective effect of miR-124 on As-induced ER stress response in neural cells (Figs. 1 and 2) and previous studies on As-induced neurotoxicity *in vitro* and *in vivo*^19–23^, we next investigated the roles of miR-124 in As-induced cytotoxicity in neural cells. First, SH-SY5Y cells were transfected with miR-124 mimic or scramble control, then exposed to As. Treatment with 1 or 5 μM As significantly decreased cell viability by ~5 or 10%, respectively, in scramble control cells (Fig. 3A). However, miR-124 overexpression by mimic transfection restored the cell viability to the level comparable to no-treatment control (Fig. 3A), suggesting a protective role for miR-124 in As-induced cytotoxicity in neural cells. To further confirm this data, we established miR-124-3 knockout SH-SY5Y cells using CRISPR-based gene editing. T7E1 cleavage assay showed that CRIPSR targeting of miR-124-3 led to efficient gene editing in the cells (Fig. 3B, Left top) as indicated by the T7E1 cleavage of reannealed PCR product (with insertions or deletions). Consistent with this, qRT-PCR showed that miR-124 expression was suppressed by more than 80% by CRISPR-based miR-124-3 knockout compared to the scrambled control (Fig. 3B, Left bottom). We then exposed control or miR-124-3 knockout cells to As, and measured cell viability. Again, treatment with 1 or 5 μM As significantly decreased cell viability by ~5 or 10%, respectively in scramble control cells (Fig. 3B, Right panel). MiR-124-3 knockout significantly exacerbated As-mediated cytotoxicity by decreasing cell viability to ~10% at 1 μM As, and ~20% at 5 μM As (Fig. 3B, Right panel), Consistent with this, indicating the protective effect of miR-124 against As-induced cytotoxicity in neural cells.

We next tested the effects of miR-124 mimics in cell models more relevant to early neurodevelopment, including human neural stem cells (hNSCs) and the neuroprogenitor ReNcell Cx cells. As shown in Fig. 3C, hNSCs were more susceptible to As exposure than the SH-SH5Y neuroblastoma cell line, with decreased cell viability to ~53% at 5 μM As. Transfection with miR-124 mimic, however, significantly increased the cell viability to ~68%. Furthermore, miR-124 mimic also improved the cell viability of ReNcell Cx neuroprogenitor cells when exposed to As (Fig. 3D) compared to scramble control. Together, these data suggest that As decreases neural cell viability potentially through increased ER stress, and that miR-124 protects against As-induced neurotoxicity.

### Association of miR-124 genetic polymorphisms with cognitive development

To further expand our *in vitro* study to human population study, we directly examined the association of miR-124 genetic variants with neurodevelopmental outcomes in children exposed to As. We utilized existing genotyping data from a genome-wide association study (GWAS) in a Bangladesh cohort^42^ that was designed to assess the effect of chronic low level As exposure on reproductive outcomes. Demographic, clinical, and neurological assessment data for the cohort are presented in Table 1.

**Table 1:** Demographic, clinical, and neurological assessment data for Bangladesh cohort SD standard deviation.

Characteristics		Mean (SD) or n (%)
		n = 502
Sex, No. (%)	Male	256 (51)
	Female	246 (49)
Concentration of arsenic in umbilical cord blood (µg/dl)^a^		0.67 (0.61)
Gestational Age, weeks		38.2 (1.7)
Age at Exam, weeks		99.4 (18.5)
Mother’s Education > Primary, n (%)		269 (54)
Smoking in Household Environment, n (%)		212 (42)
BSID Scores	Mental Composite	112.7 (10.5)
	Motor Composite	92.7 (5.0)

^a^There were 23 subjects in the Bangladesh cohort who did not have any recorded values for arsenic concentration.

**Figure 4:**
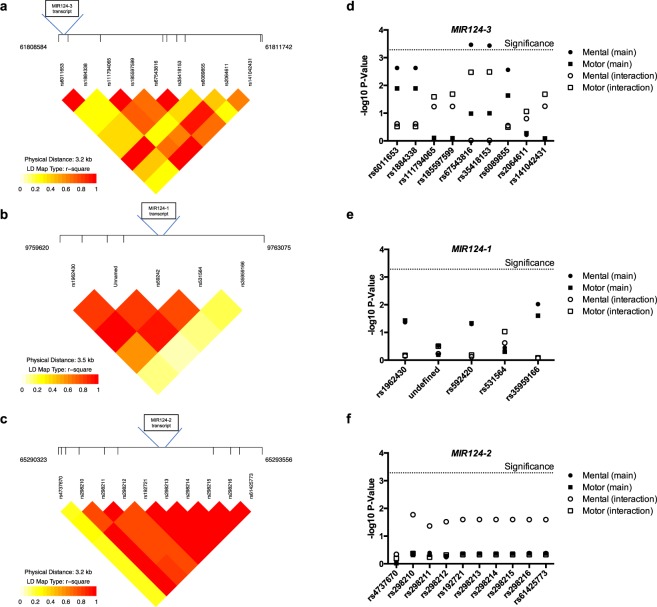
Association analysis of miR-124 SNPs with neurodevelopment phenotypes. (**a–c**) Schema of SNPs in relation to the genomic locus of miR-124-1, −2, and −3. Linkage disequilibrium (LD) patterns around miR-124 in the study population are shown. LD plot reflects pairwise R^2^ among SNPs. (**d–f**) Negative log10 p-values for SNP associations with the mental composite score (Mental) and motor composite score (Motor) as a main effect and in interaction with As in the cord blood. Subjects = 479.

The relative genomic locations and linkage disequilibrium (LD) of the miR-124 SNPs are shown in Fig. 4A–C. In main effects analyses of individual SNPs, we found significant associations between mental composite score and two SNPs in miR-124-3 (Table 2 and Fig. 4D): rs67543816 (p = 0.0003) and rs35418153 (p = 0.0004). Holding constant all of the other covariates in the regression model, each additional copy of the minor allele (C) of rs67543816 was associated with a decrease in mental composite score of 2.47. Similarly, each additional copy of the rs35418153 minor allele (G) was also associated with a decrease in mental composite score of 2.47. However, these two SNPs did not show a significant interaction with As exposure. Region-based association testing revealed a significant association between mental composite score and the main effects of SNPs in miR-124-3 (Table 3). However, this region did not show a significant interaction effect for mental composite score. There were no significant associations between SNPs in miR-124-1 or −2 and mental/motor scores (Fig. 4E,F).

**Table 2:** Summary of miR-124 SNP association effect sizes and p-values in determining mental and motor composite score^a^.

Gene	RS id	Position	Alleles	MAF	Mental composite score				Motor composite score			
					Main effect		Interaction		Main effect		Interaction	
					β	*p*-value	β	*p*-value	β	p-value	β	*p*-value
*MIR124-1*	rs1962430	9759620	G/T	0.18	1.74	0.0429	0.47	0.7109	0.85	0.0370	0.26	0.6703
	undefined	9759993	G/T	0.04	1.64	0.2958	1.13	0.5852	−0.35	0.6373	−1.00	0.3123
	rs592420	9760420	A/G	0.21	1.62	0.0495	0.39	0.7496	0.78	0.0467	0.26	0.6532
	rs531564	9760699	G/C	0.07	1.31	0.3562	2.94	0.2390	0.46	0.4957	2.00	0.0929
	rs35959166	9763075	GTGA/G	0.83	−2.33	0.0095	−0.25	0.8464	−0.96	0.0248	−0.14	0.8245
*MIR124-2*	rs4737670	65290323	G/C	0.06	−0.19	0.9018	−2.20	0.4567	0.04	0.9507	−0.66	0.6384
	rs298210	65290369	A/G	0.77	0.61	0.4259	−2.99	0.0169	0.26	0.4787	−0.48	0.4239
	rs298211	65290439	G/C	0.71	0.59	0.4044	−2.66	0.0432	0.24	0.4722	−0.35	0.5777
	rs298212	65290650	C/T	0.70	0.40	0.5755	−2.86	0.0303	0.22	0.5220	−0.44	0.4820
	rs192721	65291053	A/T	0.77	0.60	0.4287	−2.80	0.0252	0.29	0.4291	−0.44	0.4649
	rs298213	65291266	A/C	0.77	0.60	0.4287	−2.80	0.0252	0.29	0.4291	−0.44	0.4649
	rs298214	65292781	T/C	0.77	0.60	0.4287	−2.80	0.0252	0.29	0.4291	−0.44	0.4649
	rs298215	65293055	T/G	0.77	0.60	0.4287	−2.80	0.0252	0.29	0.4291	−0.44	0.4649
	rs298216	65293195	C/G	0.77	0.63	0.4084	−2.79	0.0255	0.29	0.4248	−0.44	0.4655
	rs61425773	65293556	GGAGA/G	0.77	0.63	0.4084	−2.79	0.0255	0.29	0.4248	−0.44	0.4655
*MIR124-3*	rs6011653	61808584	A/G	0.72	−2.15	0.0024	−1.06	0.2426	−0.84	0.0129	0.45	0.3042
	rs1884338	61808730	A/C	0.72	−2.15	0.0024	−1.06	0.2426	−0.84	0.0129	0.45	0.3042
	rs111794065	61809230	G/A	0.11	0.28	0.7961	−4.75	0.0581	−0.14	0.7817	−2.66	0.0257
	rs185597599	61810202	C/T	0.10	0.20	0.8574	−4.90	0.0573	−0.13	0.8037	−2.85	0.0206
	rs67543816	61810264	C/T	0.35	−2.47	***0.0003***	0.05	0.9629	−0.54	0.1033	1.44	0.0034
	rs35418153	61810418	G/A	0.34	−2.47	***0.0004***	0.05	0.9639	−0.54	0.1012	1.45	0.0033
	rs6089855	61810547	A/G	0.72	−2.11	0.0027	−0.98	0.2804	−0.76	0.0231	0.43	0.3186
	rs2064611	61811720	A/G	0.37	0.35	0.6065	−1.24	0.1573	−0.20	0.5269	−0.71	0.0865
	rs141042431	61811742	CAGT/C	0.10	0.17	0.8779	−4.92	0.0564	−0.12	0.8173	−2.84	0.0208

^a^Alleles are written in the format minor allele/major allele. A Bonferroni-corrected significance threshold of 0.05/96 = 0.00052 was used for individual SNP tests of association.

**Table 3:** Association of miR-124 SNP with infant mental and motor composite score in Bangladesh cohort^a^.

Gene	Chromosome	Number of SNPs	*P*-value			
			Mental composite score		Motor composite score	
			Main effect	Interaction	Main effect	Interaction
*MIR124-1*	8	5	0.0400	1	0.0372	0.5282
*MIR124-2*	8	10	1	0.0423	1	1
*MIR124-3*	20	9	***0.0011***	0.1668	0.0644	0.0171

^a^Using the Generalized Higher Criticism statistic, we aggregated the individual SNP test statistics for main effects and interaction for a total of 12 additional tests. A Bonferroni-corrected significance threshold of 0.05/12 = 0.0042 was used for region-based tests of association.

## Discussion

Exposure to As poses a major public health concern, especially to children, potentially affecting early brain development. Although multiple epidemiologic studies in children and animal studies have reported the adverse impact of As exposure on neurobehavioral and cognitive functions^1,2,4–10,43^, the molecular mechanisms by which As impairs neural functions remain poorly understood. In this study, we proposed ER stress as a potential mechanism for As-induced neurotoxicity and investigated the roles of miR-124, a previously identified ER stress suppressor^35^ in the regulation of As-induced ER stress in neural cells. We showed that miR-124 suppresses As-induced ER stress and cytotoxicity in neural cells including primary human neural stem cells. We also found association of miR-124-3 genetic polymorphisms with cognitive development in children. By uniquely combining the power and advantages of *in vitro* mechanistic studies in human brain cells, and human genetic epidemiologic studies in children, our study revealed a potential link between As-induced ER stress and neurodevelopment in children and suggested miR-124 as a potential therapeutic target against As exposure.

Effects of As on the protein folding in the ER represent a novel and potentially unifying mechanism that underlies the variety of toxicity associated with As exposure. With respect to neurobehavior, As-induced accumulation of misfolded proteins in the ER would lead to ER stress and subsequent activation of UPR to impact neuronal survival and death. Indeed, excess ER stress and UPR have emerged as an important mechanism for As-mediated adverse neurological outcomes^19–23^. Our study showed that miR-124, which is the most abundant microRNA in the brain and plays important roles in neural differentiation and proliferation^36^, protects against As-induced ER stress/UPR and toxicity in primary neural stem cells (NSCs). NSCs are the progenitor cells of the CNS and they play crucial roles in early brain development. Disruption of their function by As exposure may lead to deficits later in life. Our data suggest that that As perturbs ER stress and UPR signaling in NSCs to affect NSC function and to impair early brain development. We further suggest that miR-124 protects against As exposure-induced perturbation to restore function and homeostasis in NSCs. Previous studies have shown that miR-124 regulates neurogenesis and neuronal differentiation by regulating translation of its target genes such as polypyrimidine tract binding protein 1 (PTBP1), small C-terminal domain phosphatase 1 (SCP1), and SRY-Box 9 (SOX9)^44–48^. Interestingly, PTBP1 and SOX9 have been associated with ER stress and UPR genes^49,50^. We hypothesize that miR-124 exerts its protective effect against As toxicity in NSCs through one or more of these targets. Further study will be warranted to identify targets through which miR-124 exerts its protective effect against As toxicity in NSCs.

Because As exposure has been associated with neurocognitive dysfunction and our *in vitro* study showed the protective effect of miR-124 against As toxicity in neural cells, we further tested the association of miR-124 variants with neurocognitive outcomes and potential interaction with As exposure in the Bangladesh cohort. We identified two SNPs rs67543816 and rs35418153, with a statistically significant main effect association with mental composite score. Although the locations of rs67543816 and rs35418153 lie outside the genomic region of miR-124-3, they may affect transcription and processing of microRNAs^51^, which may lead to functional consequences. Although our *in vitro* studies indicates a neuroprotective role of miR-124 against As toxicity, we did not identify statistically significant interaction between the miR-124 SNPs and As exposure. Because detecting gene and environmental interactions requires a lot larger sample size in general, the Bangladesh cohort in this study presumably do not have enough power to detect such interactions. Further epidemiological studies with larger sample size are warranted to identity potential causal variants in miR-124-3 that regulate neurodevelopment in children exposed to As.

Although numerous studies have demonstrated that the mechanisms of As toxicity occur through induction of ER stress^19–23,36^, we acknowledge that ER stress may not be a main or sole mechanism for As effects on neural cells. Furthermore, due to pleiotropic roles of microRNAs, the protective effect of miR-124 in As-treated neural cells may result not only from suppressing ER stress response, but also from affecting multiple genes/pathways. For example, it was recently reported that As dysregulated REST (RE1-Silencing Transcription factor)/NRSF (Neuron-Restrictive Silencer Factor) and embryonic neural stem cell differentiation in mice potentially through interaction with miR-124^52^. Nevertheless, our study specifically addresses the unique yet less studied role of ER stress/UPR as a mechanism of As toxicity. Furthermore, we extended our mechanistic findings to human population studies by investigating the role of genetic polymorphisms of miR-124 in individual susceptibility to As exposure in a well-established child cohort. Although we identified two miR-124 SNPs that are significantly associated with neurocognitive outcomes, further study will be warranted to determine how these SNPs affect expression or function of miR-124. In addition, studies on the functional consequences of miR-124 genetic variants in neural stem cells (proliferation, self-renewal, and differentiation) against As exposure will be needed. Finally, after thorough assessment of the roles of miR-124 variants on As-induced response and neural stem cell function *in vitro*, we may extend our study to animal models for further interrogation of roles of miR-124 variants in early brain development against As exposure.

## Conclusions

In summary, we found that miR-124 protects against As-induced ER stress and toxicity in neural cells and that its genetic polymorphisms are associated with neurocognitive outcomes in children. Our study implicates a potential mechanistic link between As-induced ER stress in neural cells and neurodevelopment in children. Furthermore, our study may ultimately contribute to the development of novel microRNA-based preventative and therapeutic strategies for As-related neurodevelopmental pathologies, and potentially, other neural diseases whose pathogenesis is related to ER stress and neural stem cell dysfunction. Further mechanistic studies will be needed to elucidate the role of miR-124 in modulating the effects of As on neural cell function and neurodevelopment.

## Methods

### Cell culture and chemicals

Human neuroblastoma SH-SY5Y cells were cultured in DMEM medium (Life Technologies) with 10% FBS and antibiotics (penicillin and streptomycin, Life Technologies). Human neural stem cells derived from NIH-approved H9 (WA09) human embryonic stem cells were purchased from Life Technologies and cultured according to supplier’s protocol. ReNcell CX Immortalized Cell Line (SCC007, Millipore Sigma) derived from the cortical region of human fetal brain tissue was cultured according to supplier’s protocol. Sodium arsenite was purchased from Sigma-Aldrich.

### Overexpression of miR-124 by synthetic mimic transfection

Cells were transfected with scramble control (AllStars Negative Control siRNA, Qiagen) or miR-124 mimic (Mature sequence of miR-124-3, Syn-hsa-mir-124-3p, Qiagen; 5 pmol/well) using Lipofectamine RNAiMAX Transfection Reagent (Thermofisher Scientific). After incubation for 4 h, the medium was replaced for fresh medium, and cells were incubated for an additional 20 h. Cells were then exposed to As for 6 h (for qRT-PCR) or 48 h (for cell viability assay). Total RNAs or cell lysates were collected for qRT-PCR and Western blot analysis, respectively.

### Establishment of miR-124-3 CRISPR Knockout cells

There are three genetic locus (miR-124-1, miR-124-2, miR-124-3 for mature miR-124. Our previous study that knockout of miR-124-3 augments As-induced ER stress and cell death^35^ in HEK293T cells. To validate this finding in neural cells, CRISPR knockout cells for miR-124-3 were generated in SH-SY5Y neuroblastoma cells using specific guides (top 2 different guides for each miR-124-3) from the pooled library list. Guides were cloned into lentiCRISPRv2 vector containing hSpCas9 cassette (Addgene) as previously described^53^. T7E1 assay was performed to determine knockout efficiency following protocols in the previous study^35^. Briefly, the genomic region harboring the target of gRNAs was first PCR amplified, subjected to denaturing and reannealing temperatures (95 °C for 2 min, ramp down at −2 °C/s to 85 °C, ramp down at −0.1 °C/s to 25 °C, and stopped at 16 °C. The T7E1 (New England Biolabs) cleavage reaction was then performed at 37 °C for 20 min. The PCR products were visualized using 2% agarose gel.

### Quantitative real-time PCR (qRT-PCR)

Expression of ER stress genes including *ERN1* (gene encoding IRE1), *XBP1s*, and *CHOP* was measured by qRT-PCR following protocols in the previous study^35^. Briefly, Total RNA was extracted using RNEasy Kit (Qiagen) and reverse-transcribed to cDNA using Oligo-dT and Superscript II Kit (Life Technologies). For microRNAs, total RNA was extracted using miRNEasy kit (Qiagen). Reverse transcription and PCR were performed using miScript PCR starter kit (Qiagen). qPCR was performed using SYBR green (Qiagen) using specific primers for each gene or miRNA. The [delta][delta]Ct method was used to compare relative amounts of transcripts. β-actin and GAPDH were used as internal control for genes while RNU6B (RNU6-2) was used as internal control for miRNAs.

### Cell viability assay

We used CyQUANT Cell Proliferation Assay (Invitrogen), which can accurately quantify the entire cell population with DNA specific dye that exhibits strong fluorescence enhancement when bound to DNA, following the manufacturer’s protocol. Briefly, cells were plated in a 96-well plate at a density of 5000 cells per well and incubated overnight at 37 °C. The next day, the cells were transfected either with scramble control or miR-124 mimic as described above. Then, the cells were exposed to As for 48 h. After removing medium from wells, plates were frozen at −70 °C. The plates were thawed at room temperature, and then we added 200 μL of the CyQUANT GR dye/cell-lysis buffer to each sample well. After incubation for 2–5 min, the fluorescence was measured using a microplate reader at 480 nm(excitation)/520 nm (emission).

### Bangladesh cohort, as exposure, and neurodevelopmental indexes

A prospective birth cohort was recruited in the Sirajdikhan and Pabna Sadar Upazilas of Bangladesh from 2008–2011. The primary objective of this cohort was to observe the effects of chronic low level As exposure on reproductive outcomes. Detailed recruitment and enrollment procedures have been described previously^42^. Participants provided written informed consent. This study was approved by the Human Research Committees at the Harvard School of Public Health, Dhaka Community Hospital, and Oregon State University. The characteristics of the cohort are provided in Table S2. Relevant to this study, prenatal As exposure was assessed from umbilical cord blood at delivery. Infant neurodevelopment was assessed at 24 months of age using the Bayley Scales of Infant and Toddler Development, Third Edition (Bayley 2005). Two primary outcome indices were derived from the assessment: a mental composite score and a motor composite score. Mental composite score was calculated by summing the cognition, expressive language, and receptive language scores. Motor composite score was calculated by summing the fine motor and gross motor scores. The final sample size was 479 mother-infant pairs in Bangladesh.

### Genotyping and imputation

Broad Genomics at the Broad Institute performed genotyping with the OmniExpressExome-8 BeadChip. Further details regarding quality control measures and correction for population stratification have been described previously^54^. To more finely probe variants in our regions of interest, we then conducted imputation around three miR-124 regions using the IMPUTE2 software and 1000 Genomes Phase 3 data as a reference panel. After imputation, data was available for 5 SNPs within 5 kb of miR-124-1, 10 SNPs within 5 kb of miR-124-2, and 9 SNPs within 5 kb of miR-124-3. We extended the regions by 5 kb on each side to study the effects of possible cis-regulatory SNPs.

### Genetic association testing

We tested the association of the 24 individual SNPs in four separate models for each SNP. First, we tested the main effect of each SNP in an additive linear regression model that used mental composite score as the outcome and adjusted for confounders including maternal education level, gestational age, infant sex, age at time of neurodevelopmental assessment, household smoking, log concentration of arsenic in umbilical cord blood (ug/dl), and the first two genotype principal component vectors. Maternal education level was coded as a binary variable reflecting additional schooling past primary school. Household smoking was also coded as a binary variable reflecting the presence of any household smokers. We performed a two-sided Wald test of the null hypothesis that the effect size of the SNP was equal to 0. We then repeated this procedure using motor composite score as the outcome. Thus, 48 main effect tests were conducted.

We next conducted tests for the interaction effect of genotype and As concentration. The same linear models for main effects analysis were used in the interaction analysis, except an additional SNP-by-As-concentration interaction term was introduced into the model. We then performed a Wald test of the null hypothesis that the effect size of the interaction was equal to 0. To account for multiplicity, a conservative Bonferroni-corrected significance threshold of 0.05/96 = 0.00052 was used for individual SNP tests of association.

### Region-based association testing

We also tested for the region-based association between our two outcomes and miR-124-1, miR-124-2, and miR-124-3. Using the Generalized Higher Criticism statistic^55^, we aggregated the individual SNP test statistics for main effects and interaction for a total of 12 additional tests. A Bonferroni-corrected significance threshold of 0.05/12 = 0.0042 was used for region-based tests of association.

### Statistical analysis

Statistical analysis for *in vitro* studies was performed with GraphPad Prizm version 6 (La Jolla, CA 92037, USA). Data were analyzed by one-way analysis of variance (ANOVA) or two-way ANOVA. If significant effects were detected, the ANOVA was followed by Tukey post-hoc comparison of means. A P < 0.05 was considered statistically different. Data were expressed as means ± SEM.

### Ethics approval and informed consent

This study was approved by the Human Research Committees at the Harvard School of Public Health, Dhaka Community Hospital, and Oregon State University. This study was carried out in accordance with the relevant guidelines and regulations. Written consent was obtained from all mothers.

## Supplementary information

Supplementary Dataset 1.
