# Supplementary material for: MicroRNA-124 Reduces Arsenic-induced Endoplasmic Reticulum Stress and Neurotoxicity and is Linked with Neurodevelopment in Children
Source: Sci Rep. 2020 Apr 3;10:5934. doi: 10.1038/s41598-020-62594-8 (PMC7125130; doi:10.1038/s41598-020-62594-8)
Supplement: Supplementary file 1 — Supplementary Dataset 1. [file 41598_2020_62594_MOESM1_ESM.pdf]

SUPPLEMENTARY INFORMATION

Roles of MicroRNA-124 in Arsenic-induced Endoplasmic Reticulum Stress, Neurotoxicity, and Neurodevelopmental Outcomes in Children

Hae-Ryung Park, Ryan Sun, Ronald A. Panganiban, David C. Christiani, and Quan Lu

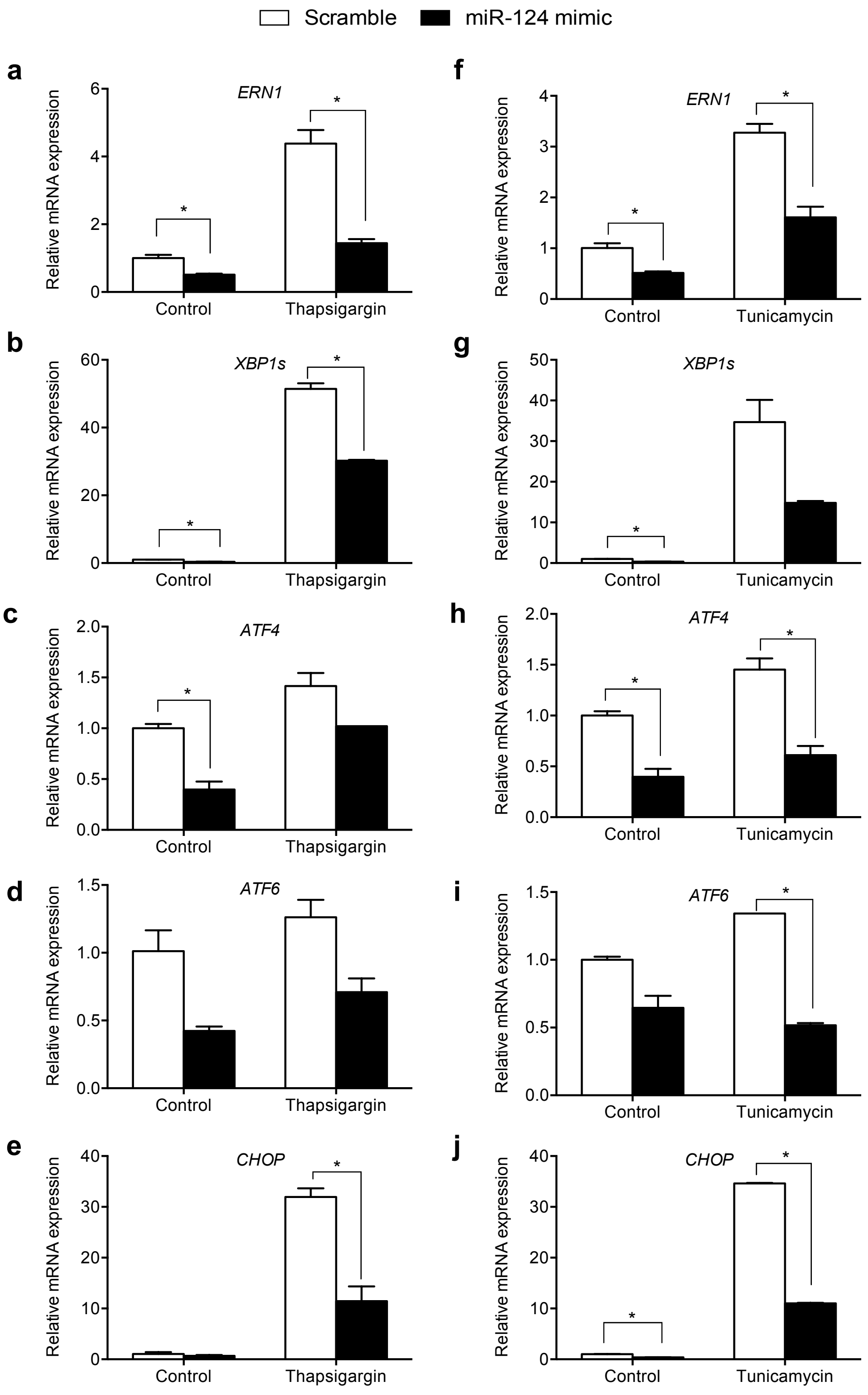

Supplementary Figure 1. MiR-124 suppresses thapsigargin or tunicamycin-induced ER stress in human neural cells. ReNcell Cx neuroprogenitor cells were transfected with scramble or miR-124 mimic, then exposed to 10 µM As for 4h. Expression of *ERN1* (gene encoding IRE1), *XBP1s* (spliced XBP1), *ATF4, ATF6* and *CHOP* was measured by qRT-PCR. N=3 experiments. All error bars represent the standard error of the mean of two biologic replicates. *, $p<0.05$

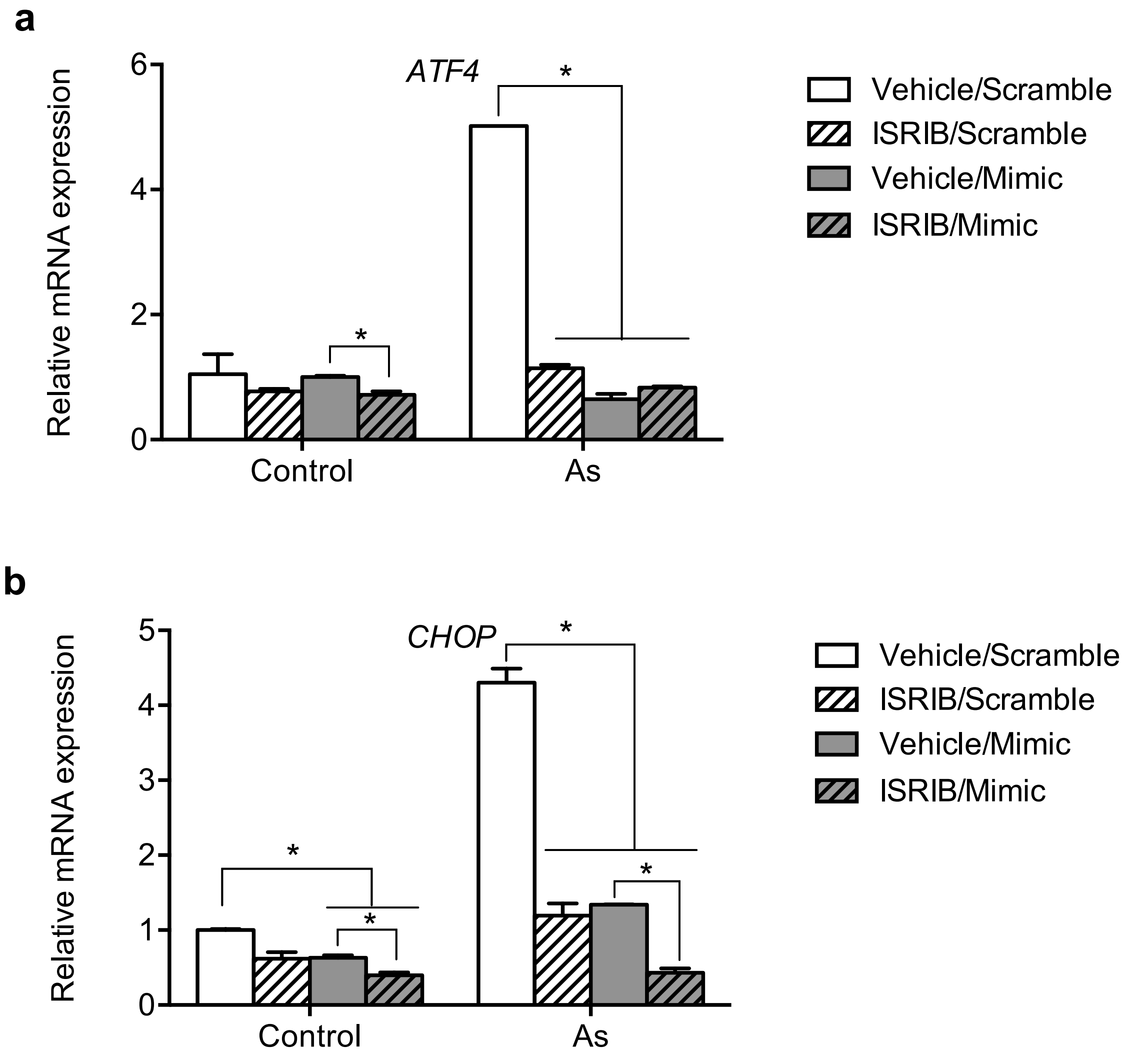

Supplementary Figure 2. Effect of PERK inhibitor on miR-124-mediated CHOP suppression. SH-SY5Y cells were pretreated with 1 μM ISRIB (PERK inhibitor) or vehicle (DMSO) for 1h, then transfected with scramble or miR-124 mimic (10 pmol/well) for 24 h. Then, the cells were exposed to As (5 μM) and mRNA expression of ATF4 and CHOP was measured by qRT-PCR. N=3 experiments. All error bars represent the standard error of the mean of two biologic replicates. *, $p<0.05$
